# Development and Analysis of an Origami-Based Elastomeric Actuator and Soft Gripper Control with Machine Learning and EMG Sensors

**DOI:** 10.3390/s24061751

**Published:** 2024-03-08

**Authors:** Meixin Wang, Wonhyong Lee, Liqi Shu, Yong Sin Kim, Chung Hyuk Park

**Affiliations:** 1 Department of Biomedical Engineering, School of Engineering and Applied Science, George Washington University, Washington, DC 20052, USA; meixin_wang@email.gwu.edu; 2 School of Computer Science and Electrical Engineering, Handong Global University, Pohang 37554, Republic of Korea; whlee@handong.edu; 3 Department of Neurology, Brown University, Providence, RI 02903, USA; liqi_shu@brown.edu; 4 School of Electrical Engineering, Korea University, Seoul 02841, Republic of Korea; shonkim@korea.ac.kr

**Keywords:** soft manipulation, artificial neural networks, highly deformable robots, soft robot, origami-based, gesture recognition

## Abstract

This study investigates the characteristics of a novel origami-based, elastomeric actuator and a soft gripper, which are controlled by hand gestures that are recognized through machine learning algorithms. The lightweight paper–elastomer structure employed in this research exhibits distinct actuation features in four key areas: (1) It requires approximately 20% less pressure for the same bending amplitude compared to pneumatic network actuators (Pneu-Net) of equivalent weight, and even less pressure compared to other actuators with non-linear bending behavior; (2) The control of the device is examined by validating the relationship between pressure and the bending angle, as well as the interaction force and pressure at a fixed bending angle; (3) A soft robotic gripper comprising three actuators is designed. Enveloping and pinch grasping experiments are conducted on various shapes, which demonstrate the gripper’s potential in handling a wide range of objects for numerous applications; and (4) A gesture recognition algorithm is developed to control the gripper using electromyogram (EMG) signals from the user’s muscles.

## 1. Introduction

Soft actuation units are increasingly garnering research interest due to their wide applicability in the biomedical field, such as in endoscopy devices, soft wearable devices, and prostheses. Their inherent advantage lies in their safe interaction with the external environment. Pneumatically powered soft actuators are particularly notable for their use of bio-compatible materials, being light weight, and delivering low-pressure actuation [1]. Pneumatic actuation also allows for complex movements with simple inputs [1,2]. Moreover, these actuators are unaffected by radioactivity and magnetic fields, thus making them suitable for magnetic resonance imaging [3]. However, a significant concern in medical applications is the “ballooning” effect, where inflation causes the actuator to expand radially, thereby increasing friction against organ walls and potentially exerting excessive pressure on tissues [4,5]. Additionally, this effect leads to energy being consumed in a radial expansion rather than longitudinal motion [4]. This optimized Pneu-Net design minimizes radial strain, dissipates significantly less energy than its original counterpart in a single actuation cycle [6], and allows for a faster actuation with less volume change for a motion amplitude of equivalent value. The wall thickness variation in actuators has also been a focus, with thicker walls requiring higher pressure for inflation [7].

Recent advancements have introduced new materials and structures. The pneumatically actuated origami chamber, which employs flexible facet transformation instead of material deformation, effectively eliminates the “ballooning” effect [8]. Origami robots offer advantages like built-in compliance due to their geometrical folds; lightweight and scalable design; and cost-effective, rapid prototyping [9,10]. A pneumatic origami actuator made from a paper–elastomer composite can achieve a wide range of motions by controlling the anisotropic response of elastomers to air pressure [11].

Pneumatically driven actuators are also prevalent in the development of soft grippers [12,13,14,15,16,17]. Unlike traditional rigid grippers, which require pre-planning for grasping objects of various geometries, soft grippers adapt passively to different shapes, thereby enabling them to grip a diverse range of objects, including delicate ones, without readjustment [18].

This study focuses on a pneumatically actuated gripper that is constructed with a silicon-paper folded structure. To assess the properties of origami-based soft actuators—including the bending angle, speed, in-plane displacement, and force generation relative to actuating pressure—a control system is necessary. However, achieving linear control remains a challenge in soft robotics. Sensory feedback is essential for control, but available flexible sensors often suffer from low sensitivity, high hysteresis, and signal drift. Liquid–metal-based soft sensors offer high sensitivity but require costly materials and complex construction [19,20]. While computer vision provides accurate position sensing, its reliance on laboratory setups can impede user mobility. Due to these sensor limitations, many soft robots operate in an open loop without camera systems [21]. Our proposed design, therefore, integrates an off-the-shelf pressure sensor that is used to estimate bending angle and force. The following table compares the present study with existing research on soft actuators, thereby highlighting our contribution to the development of the field by providing a valuable soft actuator model and its application as a compliant gripper.

This paper presents the following contributions: (i) the development and performance characterization of a soft origami-based actuator; (ii) a linear control system for regulating applied tip force; (iii) a gripper comprising three soft actuators, as well as its performance in grasping tests on various objects; and (iv) a machine-learning-based algorithm for EMG signal-controlled gripping.

## 2. Actuator Design and Characterization

### 2.1. Concept and Geometric Design

**Figure 1:**
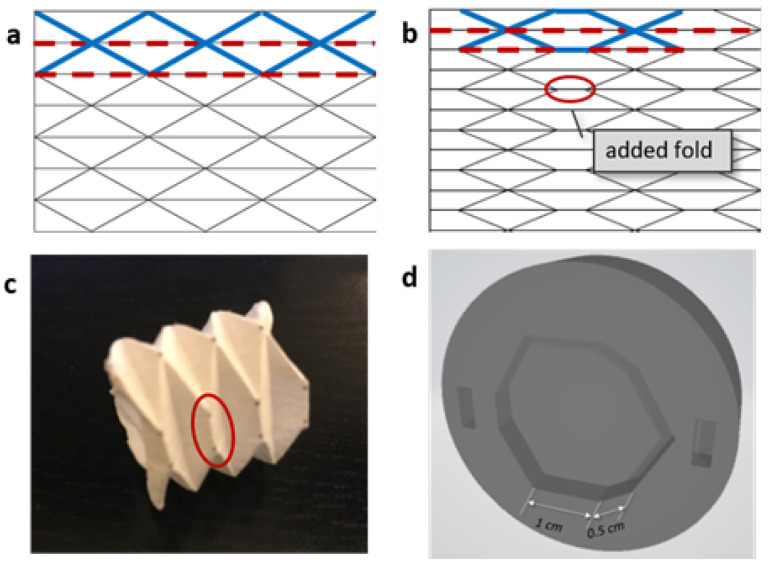
The crease pattern for the Yoshimura pattern (**a**) and the modified Yoshimura pattern (**b**), where the valley folds are shown as solid, blue lines, and the mountain folds are shown in dashed red. (**c**) The patterned paper was rolled to form a foldable cylinder, with the added fold shown in the red circle. (**d**) The mold for the cap showing a cylinder dimension in the cross section.

The proposed actuator chamber was designed by investigating and combining several primitive origami patterns to achieve the desired behavior. Existing studies on origami patterns and their modeling [22,23,24,25,26,27] have highlighted four basic patterns that are commonly used in engineering applications: the Yoshimura pattern (Figure 1a), the Miura-ori pattern, the waterbomb pattern, and the diagonal pattern. The structure design in this study was assisted by an online origami simulator [28], which generates 3D structure and strain analysis from 2D patterns. A modified Yoshimura pattern (Figure 1b) was employed in the proposed soft gripper, one that has been previously investigated for the worm robot [24]. The fold in the red circle between the two intersecting folds (Figure 1c) adds a flat surface to the folded structure in the bending direction, thus providing a larger contact area when the actuator is used as a finger of the gripper. The subsequent modeling and experiments are also based on this modified Yoshimura pattern.

**Figure 2:**
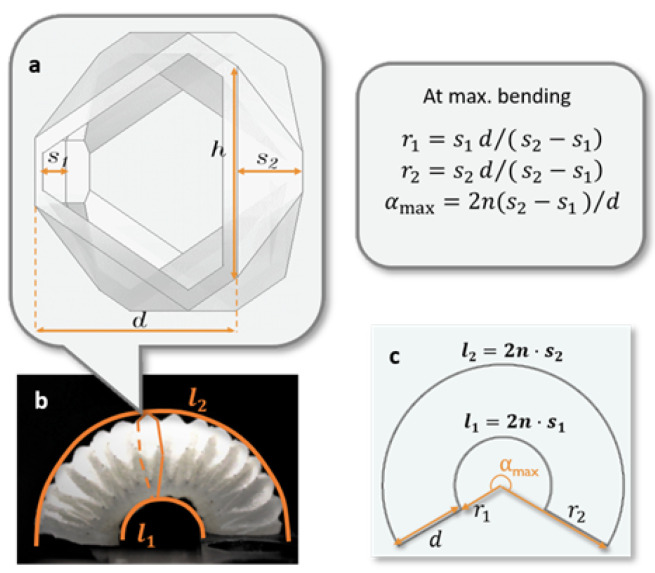
Actuator parameterization. (**a**) Dimensional parameters of one pleat; (**b**) enveloping curves of the inner and outer pleats; and (**c**) definition of the actuator’s workspace at maximum bending.

The bending behavior of the proposed actuator can be characterized by the geometrical parameters of one pleat (Figure 2a). The symbol meanings are explained in Table 1. The enveloping curves of the inflated actuator chamber (Figure 2b) were assumed to be concentric, with curve lengths of $l_{1}=2ns_{1}$ and $l_{2}=2ns_{1}+d\alpha$. Here, $l_{1}$ is constant, and $l_{2}$ is linearly associated with the evolving angle in radians, α. The actuator at maximum bending is represented as a ring shape (Figure 2c), where the inner radius $r_{1}$, the outer radius $r_{2}$, and the maximum bending angle $\alpha_{max}$ are defined by the dimensional parameters. By choosing these parameters appropriately, the desired bending range can be achieved. In this design, the dimensions were selected, as shown in Table 1, to achieve $r_{1}=10$ mm, $r_{2}=25$ mm, and $\theta_{max}=\alpha_{max}\cdot \pi /180^{\circ }=366^{\circ }$.

#### Fabrication of Actuator

**Figure 3:**
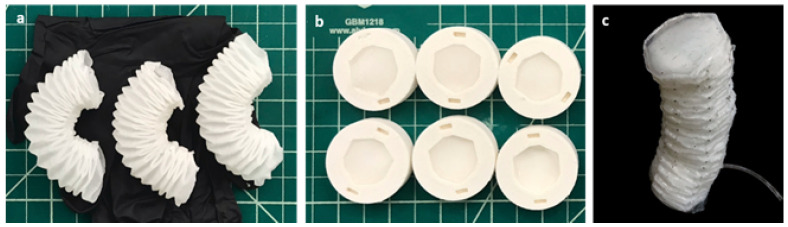
The fabrication process: (**a**) the patterned paper was rolled to form foldable cylinders, and it was then embedded in elastomer with one side of each pleat glued; (**b**) the molds for the top and bottom caps; and (**c**) the cylinder was glued with caps and was connected with an Ecoflex strip to form a pneumatic chamber.

Inspired by a soft robotic extensor based on pleated structures [11], the actuator was constructed using a folded paper structure (Spec-Wipe 5 Wiper, VWR, Radnor, PA, USA), and it was embedded in an elastomer (Ecoflex 00-30, Smooth-on, Inc., Macungie, PA, USA). Figure 3 describes the fabrication process. The modified Yoshimura pattern was used to form the foldable cylinder, which collapses under axial loading. The cylinder was evenly coated with degassed Ecoflex until saturated. It was then cured at 70 °C for 1 min. Subsequently, the partially cured structure was fully folded and held with paper clips before being completely cured at 70 °C for 20 min. One side of each pleat was glued with Ecoflex to constrain the elongation, thereby causing the actuator to bend toward that side when pressurized (Figure 3a). The top and bottom caps (Figure 3b) were made of a paper–elastomer composite, which was connected with an elastomer strip. The cylinder was sealed with these caps to form a pneumatic chamber (Figure 3c), which extended when the pleats unfolded under pressurization. The elastomer strip connecting the top and bottom caps provided a restoring force to fold the structure when depressurized. The fabricated actuator weighed a total of 9.3 g.

In the fabrication process, we initially utilized a manual approach where the paper was hand-folded and then evenly coated with Ecoflex. While this method offered simplicity and cost effectiveness, it presented challenges in achieving precise control over the folding angles and in ensuring that the Ecoflex followed the same angles as the folded paper. To address these challenges and improve the precision of our fabrication process, we explored the use of 3D flexible printing. This technique simplifies the manual fabrication process and reduces the leakage issues common in hand-made models by providing a more controlled and consistent replication of the designed folding angles. However, it is important to note that the current resolution of our 3D printer limited the minimum wall thickness of the cavity to 1.3 mm, which posed a challenge in eliminating the inner supporting material. Our ongoing efforts were focused on refining these fabrication methods to enhance the precision and scalability of our origami-based actuators.

### 2.2. Characterization

**Figure 4:**
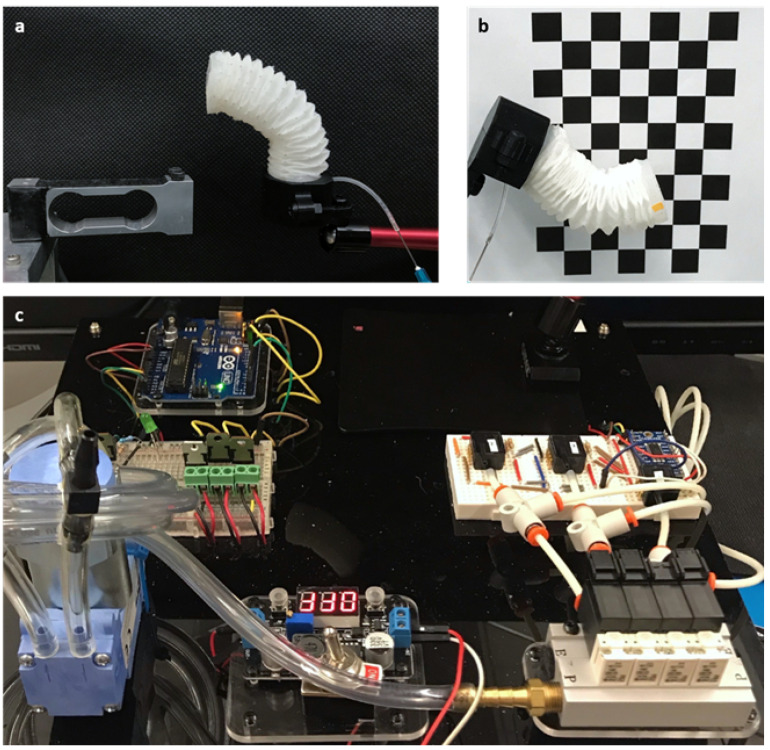
Experimental setup. (**a**) When inflated, the actuator bends toward the load cell, which measures its exerted tip force upon contact with the sensor; (**b**) in free-space bending, a camera records the tip trajectory by tracking a marker, and the checkerboard background aids in camera calibration and alignment; and (**c**) the pressure control board regulates air flow from the pump using valves and provides feedback from the pressure sensors. In the upper left, we have the Arduino microcontroller, which served as the brain of the operation. The upper right features the actuator, the primary component responsible for movement. In the middle left, there is a switch to control the pump’s operation, and adjacent to it on the middle right is the pressure sensor, which is crucial for monitoring the actuator’s internal pressure. The pump, located at the lower left, provided the necessary airflow for actuation. Directly above the pump in the lower middle is the control board’s on/off switch, which powered the system. Finally, the lower right houses the solenoid valve, which was key for directing airflow within the system.

To demonstrate the actuator’s characteristics upon pressurization, tests were conducted on an evaluation platform. These tests utilized multiple sensing modalities: a load cell (Figure 4a), a high-definition camera positioned perpendicular to the actuation plane (Figure 4b), and a pressure control system comprising a diaphragm pump, solenoid valves, and pressure sensors (Figure 4c). The associations between the pressure–bending angle, pressure displacement, and pressure force are discussed below.

#### 2.2.1. Pressure–Bending Angle Association

**Figure 5:**
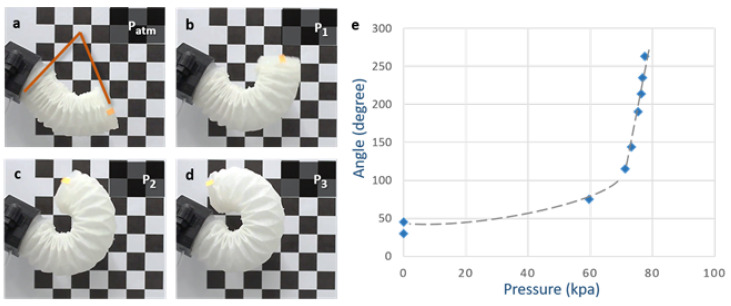
Origami-based actuators at different bending states: (**a**) natural resting position, (**b**) P1 = 71.2 kPa, (**c**) P2 = 75 kPa, (**d**) P3 = 77 kPa, and (**e**) the angle–pressure curve of the origami-based module.

With the actuator’s end clamped in a rigid fixture, the bending angle was measured as the angle between the tip and horizontal surfaces (Figure 5a). Movement in the actuation plane was recorded and analyzed using OpenCV. Compared to the “S”-shaped pressure–bending angle curve of a soft module of equal size that actuates a semi-circular chamber [4], the origami-based module exhibited the following: (i) a larger range of motion (nearly 360° with appropriate geometry); (ii) lower pressure requirements for certain bending amplitudes; (iii) smooth and continuous bending motions, with a mostly linear relationship between bending angles and pressure, from natural resting to fully bent positions. These results enabled bending angle estimation by monitoring the pressure.

#### 2.2.2. Pressure–Displacement Association

**Figure 6:**
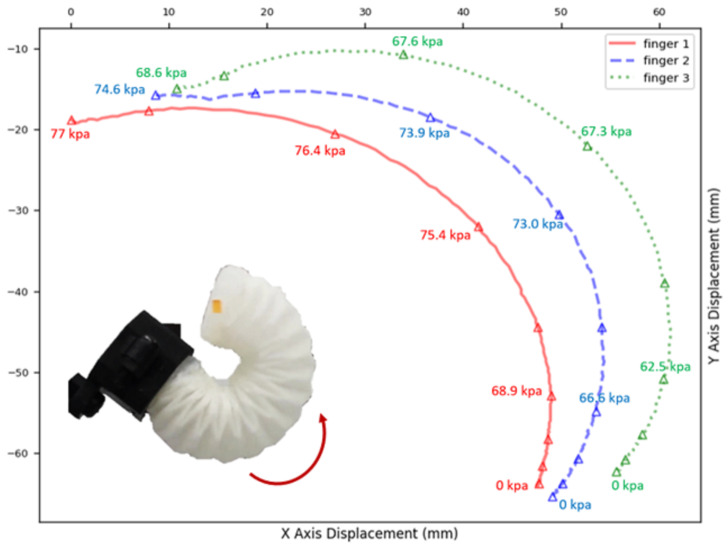
The X–Y displacement curves for three actuators under varying pressures.

Tip displacements under various pressures were recorded by tracking a marker attached to the actuator. To assess the variation among the actuators impacting the performance of a three-finger gripper, three actuators with identical folding patterns were tested. They were powered by the same pump at a constant flow rate. As Figure 6 shows, the tips of the three actuators followed similar trajectories, wherein they rapidly moved when the pressure exceeded 65 kPa and reached maximum bending at around 70 kPa. Notably, Finger 1 required relatively higher pressure, thereby resulting in a path with smaller curvature. Despite identical fabrication processes, manual steps such as the uneven thickness of silicone rubber and the dislocation of glued pleats inevitably led to discrepancies.

#### 2.2.3. Pressure–Force Association

**Figure 7:**
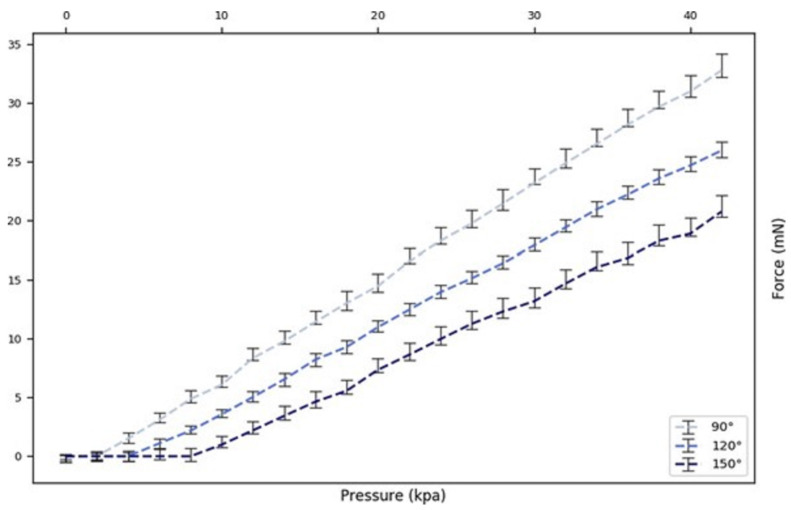
Force vs. pressure relationships at different bending angles.

The tip force was measured at different pressures while positioning the actuator fixture at angles of 90°, 120°, and 150° to the load cell. To measure the fingertip force, the actuator’s end was fixed, and the tip was slowly bent downward as the pressure increased. The tip force read zero until the actuator reached a curvature that enabled contact with the scale. The tip force vs. actuation pressure curve (Figure 7) showed an approximately linear relationship, with the slope coefficient increasing as the bending angle decreased. Thus, by knowing the bending angle at load, the pressure reading could provide a real-time force estimation without a force sensor. The bending angle at the point of contact, which was determined by observing free-load pressure changes according to the pressure–angle association, formed the basis for the gripper state.

### 2.3. Discussion

When characterizing the actuator’s bending performance, as discussed in Section 2.2, we acknowledged the significance of a theoretical model in terms of complementing these findings, even while our empirical tests provided valuable insights into the soft fingers’ functionality. The fabrication and design of our soft actuators involved a complex interplay between electrical, pneumatic, and mechanical components, thus presenting challenges in developing a mathematical model that can capture the nuances of their behavior. The robot’s continuous deformation in response to compressible gas pressure and the dynamic reaction forces during surface contact were particularly difficult to represent mathematically.

To bridge this gap, we began to explore simplified models to represent the actuator for simulation purposes. We opted to abstract the actuator’s structure into a cylindrical form, thus allowing us to formulate approximations of the kinematic and dynamic relationships. This facilitated the commencement of simulations and control systems that rely on empirical analysis, which, while preliminary, have proven to be valuable for understanding the fundamental behaviors of the soft fingers under various actuation conditions. Moving forward, we aim to refine our modeling approach by incorporating finite element method (FEM) simulations that can more accurately predict the bending behavior and mechanical performance under a range of conditions. This development will be elaborated in Section 2.2, and we will offer a comprehensive view of both our empirical and theoretical analysis methods.

In Figure 6, we document the X–Y displacement curves for the three actuators under varying pressures to investigate the variance between the actuators as this impacts the performance of a three-finger gripper. It was noted that, while the three actuators possessed identical folding patterns and were powered by the same pump at a constant flow rate, slight variances in the pressure–displacement relationships were observed. This highlighted a limitation in the current fabrication process, where manual steps such as the application of silicone rubber and pleat alignment can introduce variations. While Figure 5 and Figure 7 present the data based on one finger, they also depict an overarching pattern that was consistent across all of the three fingers. The pressure–angle relationship presented in Figure 5 and the force–pressure relationship in Figure 7 show trends that were universally observed in all of the prototypes. To address the variations observed in Figure 6, additional steps in quality control during the fabrication process were considered for future iterations to ensure more consistent actuator performance. This will be crucial for applications requiring the synchronized actuation of multiple fingers.

## 3. Gripper Testing and Gesture Recognition

A gripper comprising three origami-based modules as fingers was fabricated. It could be attached as an end effector to a manipulator using a connector. Each actuator was connected to a pneumatic source through a 0.06″ OD tube (McMaster, Elmhurst, IL, USA, Miniature EVA Tubing) and a syringe.

### 3.1. Object Gripping Capacity

**Figure 8:**
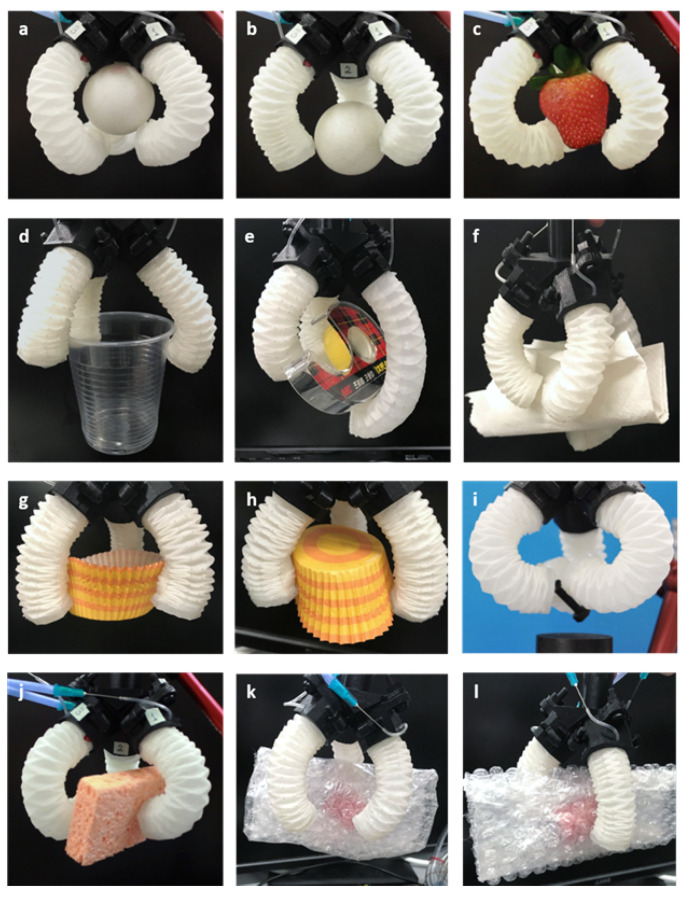
The gripper holding objects of various shapes, weights, and surfaces: (**a**) plastic cup, (**b**) tape, (**c**) screw, (**d**) paper cup downwards, (**e**) paper cup upwards, (**f**) cylinder, (**g**) paper, (**h**) weight in bubble wrap with bubbles outward, (**i**) weight in bubble wrap with the smooth side outward.

The grasp tests demonstrated the gripper’s capability in picking up objects with various geometries. Two types of grasps were tested: enveloping grasps, where objects entirely contact the gripper’s inner surface; and pinch grasps, where objects are held by the tips of the fingers. In the enveloping grasp test, objects such as a ball, a cup, and paper cups were contained within the fingers and palm (Figure 8a,d,e,g,h). Pinch grasp tests were performed on smaller or thinner objects, including a ball, a strawberry, and a sponge (Figure 8b,c,f,i,j), which could not be firmly held in the palm. The allowable load in pinch grasps was relatively lower compared to the enveloping grasps. The gripper can pick up and hold various objects without damaging delicate surfaces thanks to the compliance of the soft material. However, slight behavioral differences between the three fingers due to the fabrication process may cause orientation changes in the lifted objects and location shifts after replacement.

**Figure 9:**
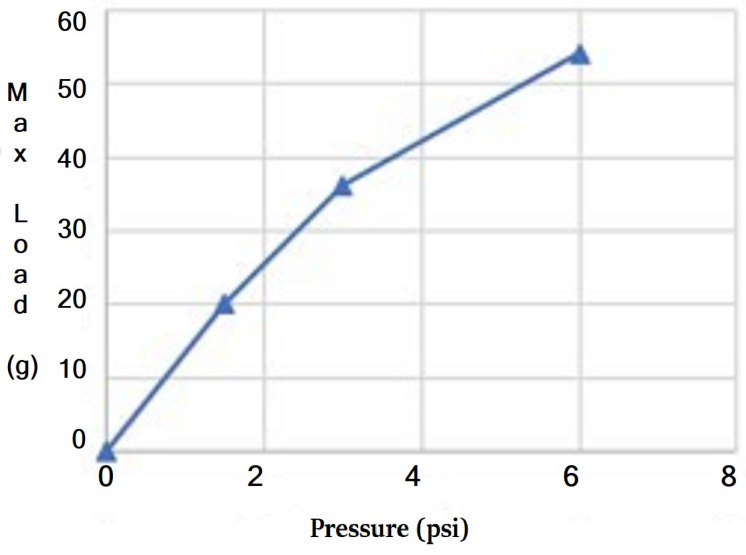
The maximum loads the gripper can hold under 1.5 psi, 3 psi, and 6 psi.

The gripper was able to lift weights of up to nearly 60 g in an enveloping grasp with the pressure limited to 6 psi to protect the thin structure. The three major factors influencing load capacity were found to be the following: (i) actuation pressure, (ii) object dimension, and (iii) object surface condition. These influences were investigated in experiments. (i) Load tests under different pressures were conducted on objects of the same shape but varying weights. Maximum loads at 1.5 psi, 3 psi, and 6 psi were tested using a cup with a 6 mm diameter opening. A direct relationship between pressure and maximum load was found and is made evident in Figure 9. (ii) Object dimensions were found to also influence load capacity. The maximum diameter of a cup-shaped object that the gripper could grasp and lift was 8 cm (almost double the range the gripper can reach in its natural resting position). This was attributed to the pleats inside the gripper holding the object’s edge. However, the gripper could not firmly hold the same cup upside down. (iii) The surface condition of the object was another critical factor. Objects with smooth surfaces are more prone to slipping. Tests on different surface conditions were performed using weights in bubble wrap, which were textured on one side and smooth on the other (Figure 8k,l). The gripper could lift twice the weight when using the textured side.

### 3.2. Contact Sensing

**Figure 10:**
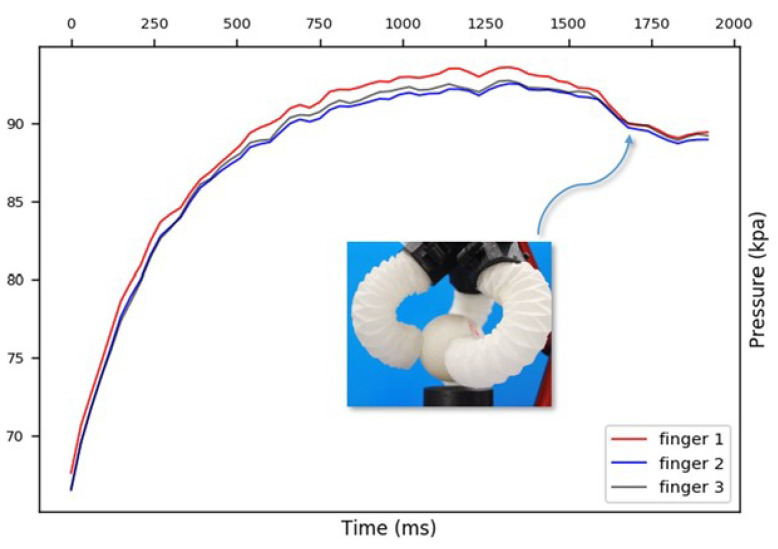
Pressure changes in the contact tests.

As demonstrated in the characterization section, the unloaded bending angle correlated with pressure readings (Figure 5), as did the interaction force with pressures at a specific bending angle (Figure 7). Thus, monitoring the pressure enabled us to gauge the grasping force, thereby knowing the extent of the actuator’s bending. Before touching an object, the actuator bends freely; if the moment of contact can be determined, the final bending angle can be inferred from the pressure reading. During three rounds of gripping tests on different objects, a non-monotonic pressure increase was observed. A significant pressure decrease occurred at the point of contact for each of the three fingers, as shown in Figure 10. Hence, with only a pressure sensor reading, both the contacting moment and the corresponding angle could be identified.

### 3.3. Actuating the Gripper with EMG Signals

Given that the actuator can fully bend in 50 milliseconds under pressurization at a rate of 24 L/min, swift operation was crucial to ensure a prompt response to user intentions. Manual input was inadequate for this requirement. The continuous and simultaneous estimation of finger kinematics was feasible using surface electromyogram (EMG) signals from the eight extrinsic muscles of the forearm, which are known to contribute to wrist and finger movements [30,31]. Therefore, the gripper was equipped with a motion sensing system where the user’s EMG signals directly dictated motion intentions [32].

**Figure 11:**
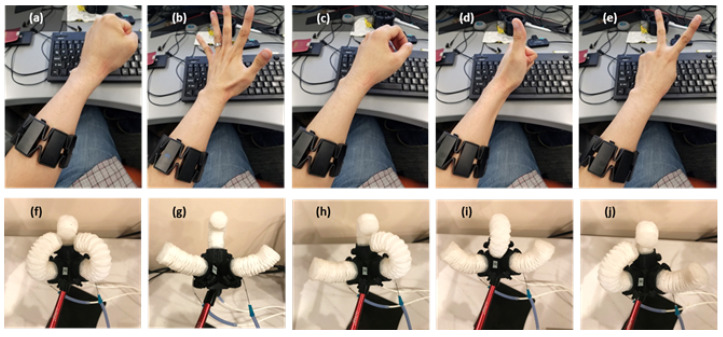
Hand gesture classification: (**a**) fist, (**b**) palm, (**c**) idle, (**d**) thumb, and (**e**) victory. The corresponding gripper motions instructed by the hand gestures are as follows: (**f**) bending three fingers, (**g**) extending three fingers, and (**h**–**j**) extending one finger. Each gesture in the upper image corresponds to a specific gripper movement in the lower image.

Eight EMG sensors were integrated into a device called the “Myo armband” (Thalmic Labs), which is worn on the left or right forearm. This device captures EMG activities from the forearm muscles at 20 frames per second. A motion indicator was developed and trained to recognize multi-channel muscle signals, as well as to classify the user’s hand gestures. The input to the motion classifier comprised eight-channel temporal data from a Myo armband on a forearm, where 1 s consecutive data were captured, as this was empirically determined to be the minimum length for meaningful EMG signals. The classifier’s output indicated the probability of five gestures (as shown in Figure 11a–e): fist, palm, idle, thumb, and victory.

**Figure 12:**
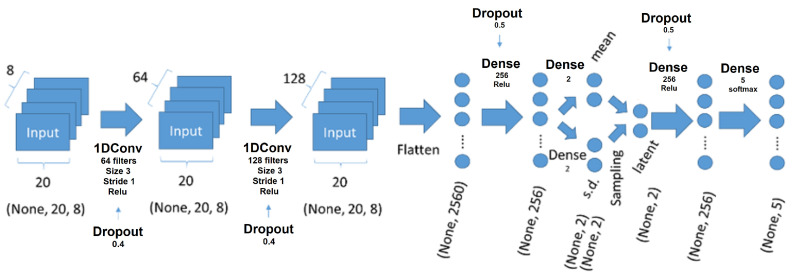
The architecture of the multi-channel and multi-layer convolutional neural network, which incorporated variational autoencoder (VAE) elements, was implemented in the classifier. This network structure was designed for the efficient and effective gesture classification of EMG data, thereby leveraging the strengths of both the 1D CNN layers for temporal pattern recognition and VAE for latent feature extraction.

A multi-channel and multi-layer convolutional neural network (CNN) incorporating variational autoencoder (VAE) elements was used to implement the temporal multi-label classifier, and it featured the following characteristics: (1) Two 1D CNN layers to identify temporal data patterns (64 and 128 filters, respectively, with a kernel size of 3 and 40% dropout). (2) Two dense layers to reduce the feature space to a latent distribution space (256 nodes in the middle layer and 50% dropout), which enables the classification of input patterns similar to but not present in the training dataset as one of the five gestures. (3) The sampling from a two-dimensional latent space was followed by two additional dense layers (256 nodes in the middle layer and 50% dropout), which provided probabilities for the five gestures. The network model is illustrated in Figure 12.

**Figure 13:**
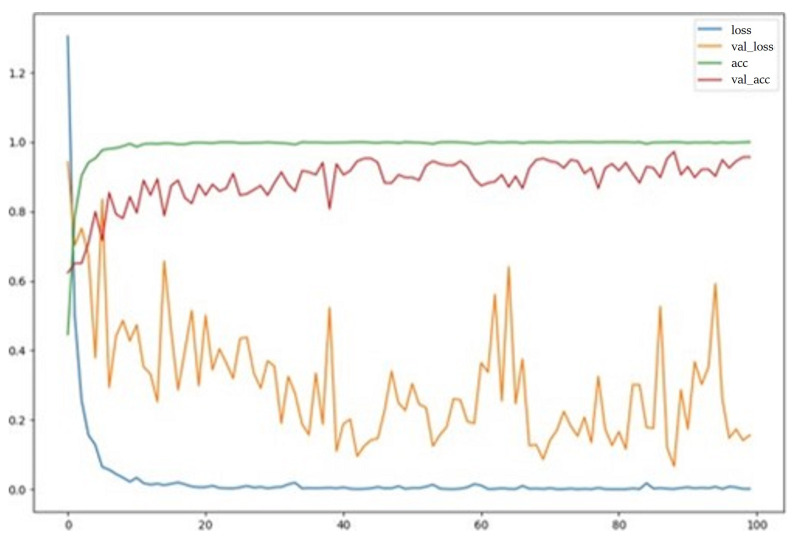
The training accuracy and loss over 100 epochs.

The model was trained using the Adam optimizer and categorical cross-entropy for over 100 epochs, and it achieved a nearly 100% training accuracy and up to 95% test accuracy, as shown in Figure 13.

The identified gesture was then used to command five types of movements in the gripper (as depicted in Figure 11f–j): complete extension, complete bending, and independent bending of each finger.

The choice of employing a variational autoencoder (VAE) and 1D CNNs in our model was grounded in both empirical evidence and theoretical suitability. The VAE framework is adept at learning the latent characteristics of high-dimensional data, such as EMG signals, whereby it effectively captures essential features through data compression. This is particularly beneficial for modeling complex EMG patterns as it enables the generation of new, unseen data that mimic the characteristics of the training set. Such an ability is crucial in adapting to diverse user EMG patterns and handling novel signals that might arise in real-world applications.

Moreover, the use of 1D CNN layers was justified due to their proficiency in learning temporal features from sequential data. This is vital for processing time series data like EMG signals, where capturing the temporal dynamics is essential. In addition, 1D CNN layers offer efficient learning with fewer parameters, thus making them ideal for real-time applications where quick processing is required.

The parameters of the model, including the number of filters, kernel size, and dropout rates, were determined through empirical methods. These were optimized through a series of experiments, wherein the aim was to find the most effective network architecture for our specific application. This process, underpinned by empirical know how, helped with evaluating the network’s performance across various datasets and conditions, thereby refining the parameters for enhanced generalization and applicability in real-world scenarios.

In summary, the integration of VAE and 1D CNN layers in our model provided a robust and flexible solution in accurately classifying the hand gestures from EMG data. This approach effectively harnesses the power of deep learning for real-time, efficient, and user-adaptive gesture recognition, thus making it highly suitable for the intended application of controlling a pneumatically actuated soft gripper.

## 4. Discussion

In this study, a lightweight soft actuator based on the Yoshimura crease pattern was successfully fabricated and characterized as in Table 2. The performance of the actuating module was assessed using four key parameters: (i) the pressure required to achieve a specific degree of bending, (ii) the time taken to actuate from a linear shape to full extension, (iii) the force exerted for a given pressure when interacting with the environment, and (iv) the linearity of the fitted curves for angle–pressure and force–pressure data.

Compared to other pneumatic soft actuators, our origami-based actuator exhibited improvements in actuation pressure, speed, and linearity relative to Pneu-Net and pneumatic devices with semi-circular chambers. The well-defined associations observed in the angle–pressure and force–pressure curves enabled straightforward and accurate linear control.

It is worth noting that, while the maximum force generated by our actuator was limited due to the tear strength of the thin paper–elastomer composite, there is a potential for improvement by increasing the stiffness of the elastomer. Future research can explore the various silicone and polyurethane elastomers available in the market, which offer a wide range of material properties. The actuator proposed in this study holds promise for small-scale medical applications, particularly in scenarios requiring forces in the range of 1 N. For instance, the bellows structure can navigate through narrow channels when folded, and it can unfold within an organ chamber to interact with tissues. Its low-pressure requirements make it suitable for tasks in biomedical environments with limited space, such as minimally invasive surgery and drug delivery.

Additionally, the actuator can serve as an end effector for delicate apparatus operations, as validated by the gripper prototype consisting of three fingers. The soft gripper demonstrated compliant gripping capabilities for objects of various shapes. Contact sensing through pressure sensors implies the potential for haptic object identification without the need for motion and vision capture devices. The prompt operation of gripping was made possible by the motion intention identifier built using EMG signals. This novel approach provided an intuitive and user-friendly method for controlling soft robotic devices, which has broad implications in the fields of biomedical applications and robotics.

However, there are limitations to our current design. The maximum force generated by our actuator is constrained by the tear strength of the thin paper–elastomer composite. Future research will explore the use of various silicone and polyurethane elastomers to improve the actuator’s mechanical performance, and work on this could be expanded by conducting a more comprehensive comparative analysis of various origami patterns, including detailed force and strain analyses under different pressure conditions.

Future work will focus on enhancing the fabrication consistency, efficiency, and mechanical performance of the actuator. This includes investigating the material stiffness and durability to further broaden its applicability in various domains.

## Figures and Tables

**Table 1:** Comparison of the different actuators.

	Actuator 1 (the Pyramid Robot) [29]	Actuator 2 (Pneu-Net) [6]	Actuator 3 (the Pleated Structure) [11]	The Proposed Actuator
**Energy source**	Electrical	Pneumatical	Pneumatical	Pneumatical
**Material**	Dielectric elastomers	Silicon rubber	Paper–elastomer composite	Paper–elastomer composite
**Pattern**	N/A	Connected chambers on an inextensible layer	Yoshimura pattern	Modified Yoshimura pattern
**Weight**	2.44 g	More than 15 g	8.3 g	~10 g
**Motion**	Bend 120°	Bend 360° and radical expansion	Bend 360° extend ϵ=3.61	Bend 360° to form a gripper
**Actuation speed**	2–3 s	50 ms when pressurized at 345 KPa	352 ms when pressurized at 60 KPa	50 ms when pressurized at 24 L/min
**Force exerted**	0.06 N	~1.4 N	Unknown	0.6 N

**Table 2:** Design parameters.

Symbol	Meaning	Value
$s_{1}$	Distance between inner pleats (glued)	2 mm
$s_{2}$	Outer pleat width	5 mm
*d*	Distance between edges of outer and inner pleats	15 mm
h	Fold length	17.3 mm
*n*	Number of pleats	16

## Data Availability

Data are unavailable due to privacy or ethical restrictions.
